# A *Candida* Biofilm-Induced Pathway for Matrix Glucan Delivery: Implications for Drug Resistance

**DOI:** 10.1371/journal.ppat.1002848

**Published:** 2012-08-02

**Authors:** Heather T. Taff, Jeniel E. Nett, Robert Zarnowski, Kelly M. Ross, Hiram Sanchez, Mike T. Cain, Jessica Hamaker, Aaron P. Mitchell, David R. Andes

**Affiliations:** 1 Departments of Medicine and Medical Microbiology and Immunology, University of Wisconsin, Madison, Wisconsin; 2 Department of Microbiology, Columbia University, New York, New York; 3 Department of Biological Sciences, Carnegie Mellon University, Pittsburgh, Pennsylvania; Washington University School of Medicine, United States of America

## Abstract

Extracellular polysaccharides are key constituents of the biofilm matrix of many microorganisms. One critical carbohydrate component of *Candida albicans* biofilms, β-1,3 glucan, has been linked to biofilm protection from antifungal agents. In this study, we identify three glucan modification enzymes that function to deliver glucan from the cell to the extracellular matrix. These enzymes include two predicted glucan transferases and an exo-glucanase, encoded by *BGL2*, *PHR1*, and *XOG1*, respectively. We show that the enzymes are crucial for both delivery of β-1,3 glucan to the biofilm matrix and for accumulation of mature matrix biomass. The enzymes do not appear to impact cell wall glucan content of biofilm cells, nor are they necessary for filamentation or biofilm formation. We demonstrate that mutants lacking these genes exhibit enhanced susceptibility to the commonly used antifungal, fluconazole, during biofilm growth only. Transcriptional analysis and biofilm phenotypes of strains with multiple mutations suggest that these enzymes act in a complementary fashion to distribute matrix downstream of the primary β-1,3 glucan synthase encoded by *FKS1*. Furthermore, our observations suggest that this matrix delivery pathway works independently from the *C. albicans ZAP1* matrix formation regulatory pathway. These glucan modification enzymes appear to play a biofilm-specific role in mediating the delivery and organization of mature biofilm matrix. We propose that the discovery of inhibitors for these enzymes would provide promising anti-biofilm therapeutics.

## Author Summary

Biofilms are a community of microbes that grow attached to each other and adherent to a surface. One distinguishing feature of this form of growth is the presence of a surrounding extracellular matrix which is proposed to provide a structural scaffold and protection for biofilm cells. This later function contributes to the extreme resistance to anti-infective therapies, another innate characteristic of biofilms. One carbohydrate component of the matrix of *Candida albicans*, β-1, 3 glucan, has been linked to overall accumulation of matrix material and the antifungal drug resistance phenotype. Although the glucan synthase pathway has been implicated in glucan production, the delivery and incorporation of these carbohydrates into the matrix remains a mystery. The current investigation describes three gene products that serve a matrix delivery role. The functions of these gene products include glucanase and glucanosyltransferase activities. Mutants unable to produce these enzymes demonstrate reduced matrix glucan, decreased total matrix biomass accumulation, and enhanced susceptibility to antifungal drug therapy. The observations here offer insight into a novel pathway that contributes to biofilm maintenance. Enzymes in this biofilm-specific process may provide useful anti-biofilm drug targets.

## Introduction

*Candida spp*. are an increasingly common cause of bloodstream infections in hospitalized patients [1], [2]. This rise in incidence is at least in part related to the organism's ability to produce biofilm infections on medical devices [3]. A biofilm is a community of microbes attached to a surface and encased in an extracellular matrix [4]–[6]. The biofilm lifestyle is a common form of growth in nature and the most common cause of infection in humans. The most troublesome characteristic of biofilms is that they are up to 1,000-fold more resistant to common antifungals than their planktonic counterparts, even without accumulation of specific drug-resistance genes [7]–[10]. This lack of effective therapy contributes to dismal outcomes for patients with invasive candidiasis, including death in up to 40% of patients. Delineating the mechanisms of biofilm formation and associated treatment resistance is one method of identifying optimal management strategies and therapeutics of this devastating infectious disease.

The focus of our investigations is the construction and configuration of the extracellular biofilm matrix, one of the properties that distinguishes biofilm from planktonic growth [11]. The function of matrix remains incompletely understood, but previous investigations have identified roles such as providing infrastructure for biofilm accumulation, controlling disaggregation, and granting protection from antimicrobial drugs and the host immune system [12]–[14]. Although the complete composition of the *C. albicans* biofilm matrix has yet to be fully elucidated, studies have identified the inclusion of carbohydrates, proteins, and nucleic acids components [11], [13], [15]. The goal of the present studies was to identify genes that control matrix delivery. We hypothesized that this process involves a biofilm-specific pathway composed of enzymes capable of modifying matrix carbohydrates. This hypothesis is based on two findings. First, one of the carbohydrates, β-1,3 glucan, has been linked to overall matrix production and drug resistance through glucan synthase gene *FKS1* (common nomenclature for the gene *GSC1*) [16], [17]. Second, microarray analysis of in vivo rat catheter biofilms demonstrated transcript abundance of multiple glucan modification genes [18].

Here we use a candidate gene set to investigate the role of glucan matrix delivery. The gene set was selected to include glucan modification genes which demonstrated transcriptional upregulation in a rat venous catheter biofilm model. In addition, we included gene products which are known or hypothesized to utilize β-1,3 glucan as a substrate [19]–[25]. Many of the selected genes had been shown previously to function in planktonic cell wall synthesis and remodeling [23]–[30]. We constructed gene mutants and screened for biofilm formation, matrix delivery and antifungal drug susceptibility.

In the current studies we describe the role of three glucan modifying genes for glucan delivery and matrix incorporation. These gene products encode two glucanosyltranferases (*BGL2*, *PHR1*) and a glucanase (*XOG1*), respectively [22], [23], [25]–[29]. Each appears necessary for modification and delivery of carbohydrate to the mature biofilm matrix. Without delivery and accumulation of matrix glucan, the biofilms exhibit enhanced susceptibility to antifungal drugs. As the biofilm matrix is integral for biofilm maintenance and drug resistance, these delivery enzymes provide promising targets for anti-biofilm drug development.

## Results

### A novel glucan modifying pathway for extracellular matrix delivery

**Figure 1:**
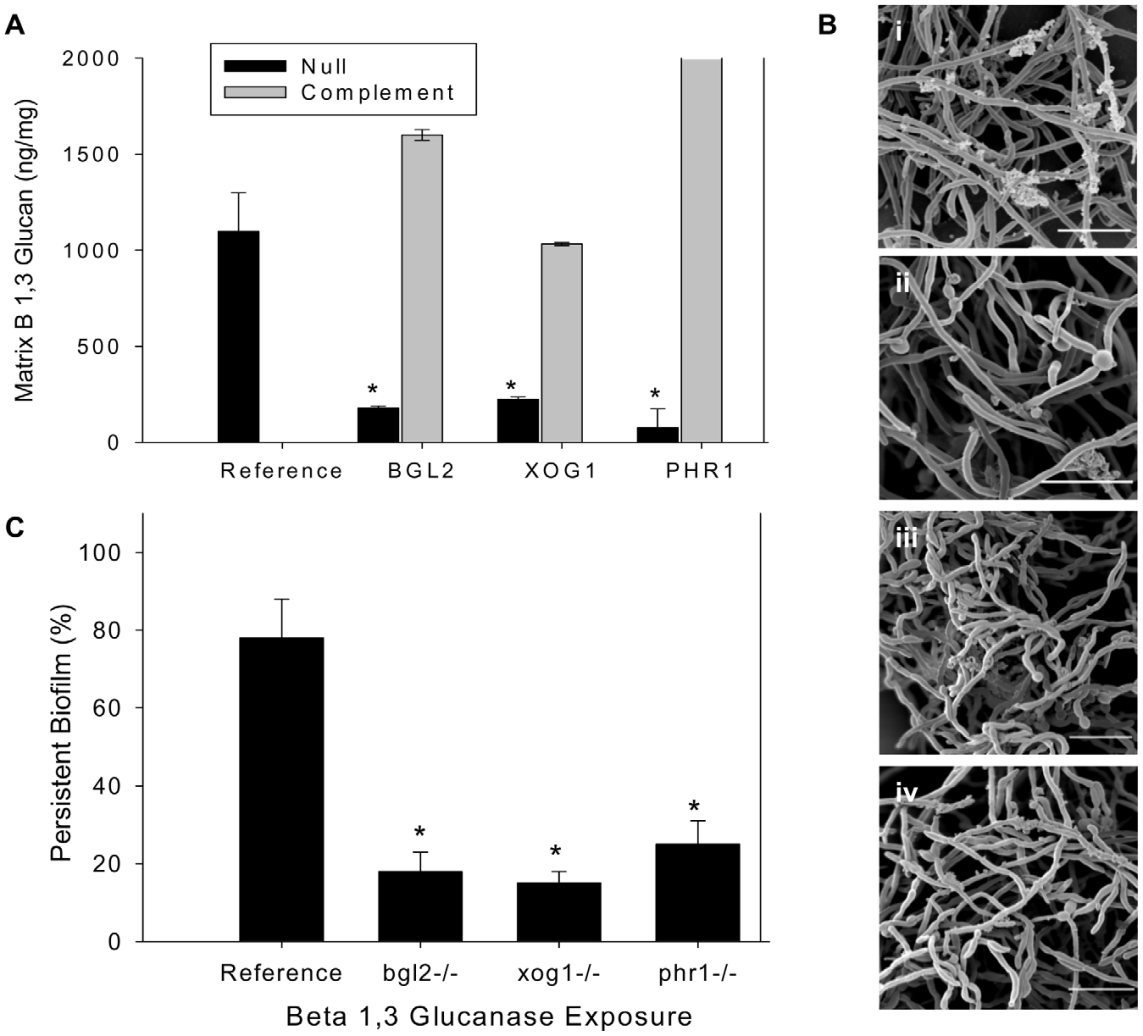
Analysis of biofilm matrix. (A) Mature in vitro biofilms from the reference strain, *bgl2*−/−, *xog1*−/−, and *phr1*−/− null mutants, and complemented strains were assayed for matrix β-1,3 glucan concentration using a β-1,3 glucan ELISA. The figure represents data from three biologic and three assay replicates. The * symbol indicates that glucan measurements were significantly different (p value<0.0001) based upon ANOVA compared to the reference strain. (B) Matrix abundance from mature in vitro biofilms produced by the reference and *bgl2*−/−, *xog1*−/−, and *phr1*−/− mutant strains was estimated visually by SEM imaging. Scale bars represent 20 µm. (C) Mature biofilm disaggregation was measured following matrix exposure to β-1,3 glucanase (2.5 units/ml) in reference and *bgl2*−/−, *xog1*−/−, and *phr1*−/− mutant strains. The non-disaggregated cells were measured using the XTT assay. The * symbol indicates biofilm biomass measurements were significantly different compared to the reference strain (p value<0.01) based upon ANOVA.

We have previously described the presence of β-1,3 glucan in the biofilm matrix of *C. albicans* and identified the role of the glucan synthase pathway for production of this material [16], [17], [30]. The machinery needed for delivery of this matrix component from the cell to the matrix was, however, not known. We reasoned that proteins that act upon a glucan substrate might contribute to the delivery process. Results of an in vivo microarray analysis of a rat venous catheter biofilm demonstrated differential expression of 11 potential glucan modification genes [18]. A candidate gene set was constructed by combining these 11 genes with 4 additional genes selected from a search of the *Candida* genome database for putative glucan modifying function (glucanases, transferases, and glucosidases). A combination of homozygous deletion mutants were created for fourteen genes and a heterozygous mutant for one gene presumed to be essential (**Table S1 in Text S1**). Our initial experiments consisted of two screens. First, we examined overall biofilm growth in all strains. Each of the mutants produced mature in vitro biofilms similar to reference strains, with the exception the *phr1*−/− strain which exhibited a modest biofilm defect (75% cell burden compared to the reference strain). The *phr−/−* strain also demonstrated a modest defect in adhesion to a polystyrene substrate (67% relative to the reference strain). The mutant strains exhibited normal planktonic growth in YPD compared to the reference strain. Secondly, we measured the β-1,3 glucan concentrations in the matrix from mature in vitro biofilms using both the commercial limulus lysate assay (Glucatell) and a glucan ELISA. These assays identified three deletion mutants, *bgl2*−/−, *xog1−/−*, and *phr1*−/−, which produced up to 10-fold less matrix β-1,3 glucan than the reference biofilm (**Table 1** **and** **Figure 1A**). The observations were confirmed in independent transformants for each gene (**Table 1**). Furthermore, complementation of the mutants with a single copy of each gene restored glucan matrix concentrations to reference strain levels.

**Table 1:** Biofilm and planktonic phenotypes of select glucan modification mutant strains.

Systematic Name	Gene Name	Genotype	Description****	In vivo transcript RT (Biofilm/Plank) 48 h	Independent Strains	Biofilm Formation*	Biofilm Resistance**	Matrix Glucan*** (pg/ml)	Planktonic MIC (µg/ml)
-	-	Reference			SN250	100%	100%	1700	0.5
					DAY185	100%	100%	3660	0.5
19.4565	*BGL2*	−/−	β-1,3 glucanosyltransferase	3.1	FB63	120%	30%	890+	0.5
					HTT111	92%	49%	1115++	0.5
19.2990	*XOG1*	−/−	β-1,3 glucanase	7.3	GKO229	96%	48%	570+	0.5
					HTT117	110%	51%	1011++	0.5
19.3829	*PHR1*	−/−	β-1,3 glucanosyltransferase	24.2	KMR101	75%	36%	190++	0.25

* Percent biofilm formation compared to reference strain.

** Percent of biofilm remaining after exposure to fluconazole at 1000 µg/ml,

*** Matrix β-1,3 glucan concentration normalized to biofilm burden,

**** Based upon Candida or Saccharomyces Genome Database.

+ Strains compared to reference strain DAY185.

++ Strains compared to reference strain SN250.

The relevance of these glucan modification genes to in vivo biofilm growth is suggested by their transcriptional abundance in a rat venous catheter biofilm [18]. At 12 h of in vivo biofilm growth, microarray studies showed that transcription of *BGL2* and *PHR1* was upregulated. During mature biofilm growth (24 h), *BGL2* and *XOG1* transcripts were abundant. RT-PCR confirmed marked increases in expression during biofilm growth (**Table 1**). We asked if these glucan modification enzymes were functioning in conjunction with the previously described Zap1-regulated matrix production [31]. This zinc transcription factor is a negative regulator of biofilm matrix production, including matrix glucan production. Surprisingly, these glucan modification enzymes appear to function independently of Zap1. First, transcription of *BGL2*, *XOG1*, or *PHR1* was not significantly altered in the zap*1−/−* mutant biofilm. Second, there were no significant changes in *ZAP1* transcription in the glucan modification mutant biofilms (data not shown). These findings suggest that *BGL2*, *XOG1*, and *PHR1* comprise a distinct biofilm matrix delivery pathway.

### Extracellular matrix delivery is critical for securing biofilm cells to a surface

The mutants with reduced matrix glucan (*bgl2−/−*, *xog1−/−*, and *phr1−/−*) were evaluated for biofilm architecture, matrix appearance, and total matrix abundance by scanning electron microscopy of in vitro biofilms. These glucan modifying enzyme mutants were capable of biofilm formation, but exhibited diminished extracellular biofilm material (**Figure 1B**). The association between reduced glucan and total matrix biomass is similar to that described for mutants in the β-1,3 glucan synthesis pathway [17], [30]–[33].

Since β-1,3 glucan has been described as a matrix component, we considered the possibility that the glucan in the matrix may also impact biofilm persistence or resistance to disaggregation. To test the functional role of matrix glucan, and the impact of glucan matrix delivery, we examined biofilm cell disaggregation in the reference strain and this subset of glucan modifying enzyme mutants following exposure to low concentrations of β-1,3 glucanase. Previous studies in this model have shown that higher concentrations of this enzyme will disperse intact mature biofilms [16]. In the present investigation, exposure to a low concentration of β-1,3 glucanase resulted in disaggregation of approximately 25% of the reference biofilm (**Figure 1C**). However, the same glucanase incubation allowed dispersion of approximately 80–90% of the glucan modifying mutant biofilms. These observations argue that matrix β-1,3 glucan provides an adhesive function within the biofilm matrix. The disaggregation findings are also consistent with the matrix biochemical and imaging observations showing less matrix β-1,3 glucan and total matrix biomass.

### Glucan modifying enzymes contribute to biofilm matrix-associated antifungal resistance

**Figure 2:**
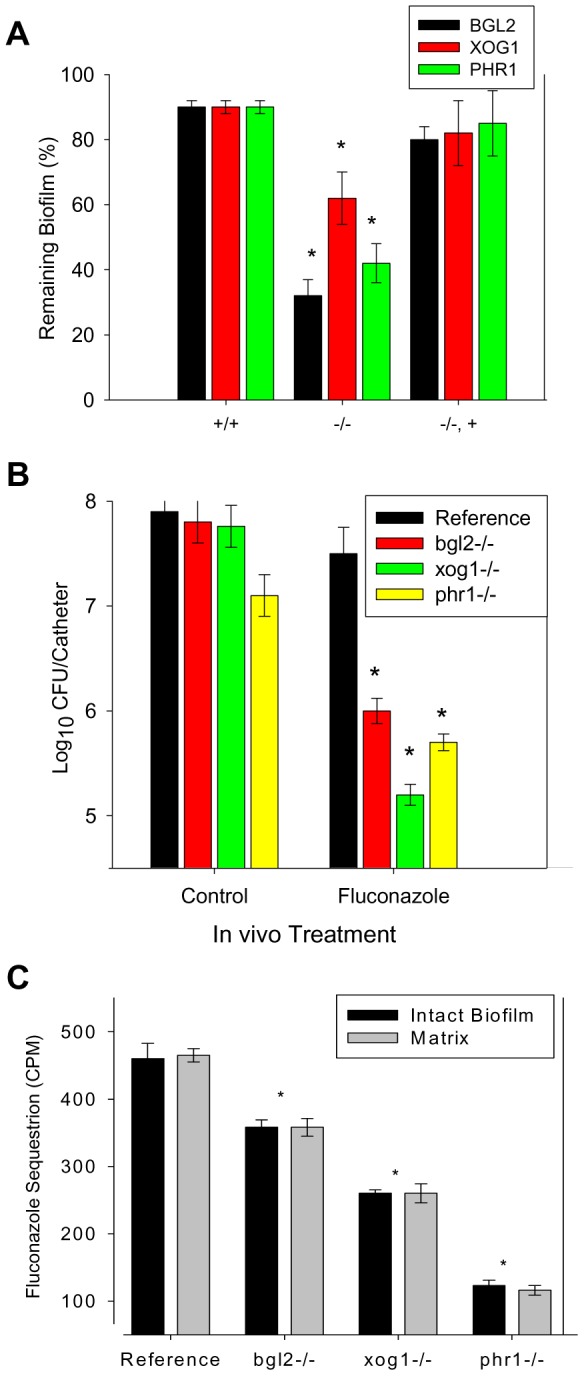
Biofilm drug susceptibility and matrix drug sequestration. (A) Mature in vitro biofilms from the reference strain and *bgl2*−/−, *xog1*−/−, and *phr1*−/− null mutants and complemented strains were assayed for fluconazole susceptibility using the 96-well XTT assay. The figure represents data from three assay replicates of a representative example of 3 biological replicates of the 250 µg/ml fluconazole exposure. (B) Mature in vivo biofilms from the reference strain and *bgl2*−/−, *xog1*−/−, and *phr1*−/− null mutants were grown on the rat central venous catheter model and then exposed to a dwell of 250 µg/ml of fluconazole or 0.15 M NaCl for 24 h. Following sonication, the burden of remaining biofilm cells was assayed using viable plate counts. The figure represents the mean and standard deviation from three replicates. The * symbol indicates CFUs were significantly different from the reference strain (p value<0.01) based upon ANOVA. (C) Intact biofilms grown from the reference and glucan modifier mutant strains were exposed to [H3]fluconazole, washed, and harvested. Scintillation counting was performed in triplicate to determine the fluconazole content in the intact biofilms and the isolated matrix. Standard deviations are shown. The * symbol indicates glucan measurements were significantly different (p value<0.03) based upon ANOVA.

A previously demonstrated link between matrix glucan and drug resistance led us to test the impact of these glucan modifying enzymes on this important biofilm phenotype [14], [16]. Each of the fifteen glucan modifying mutants in the candidate gene set was screened for susceptibility to the triazole, fluconazole, during in vitro biofilm growth (**Table 1** **and Table S1 in Text S1**). The three glucan modifying mutants that delivered less matrix glucan exhibited enhanced susceptibility to fluconazole. Although the highest concentration of fluconazole resulted in no net change in cell burden in reference biofilms, this same drug exposure reduced the mutant biofilms by 35 to nearly 70% (**Table 1** **and** **Figure 2A**). A dose dependent anti-biofilm effect was observed over the entire dose range examined (not shown). These findings were confirmed for the independent transformants (**Table 1**). Furthermore, the biofilm-associated antifungal resistance was restored in complemented strains (**Figure 2A**). Deletion of the three glucan modifying genes did not cause a significant change in planktonic antifungal drug susceptibility (**Table 1**), so we infer that this is a biofilm-specific phenotype.

As drugs from the echinocandin class target β-1,3 glucan synthesis, we further examined the impact of these select glucan modification mutants on biofilm susceptibility to a drug from this class. Each of strains (parent and the three mutants) demonstrated extensive susceptibility to low echinocandin concentrations (<0.03 µg/ml). No difference in drug activity was observed over the range of concentrations examined.

In order to determine the clinical relevance of these observations, we examined drug susceptibility using the in vivo rat central venous catheter biofilm model [34]. The impact of the fluconazole treatment was tested by measuring the viable burden of biofilm cells present following a twenty-four hour period of exposure to the drug instilled within the catheter lumen. Drug treatment produced minimal change in biofilm burden in the reference strain. In vivo study of the glucan modifying mutants recapitulated the observations from the in vitro model. The burden of catheter associated cells was reduced by 1.5 to more than 2 logs compared to the reference strain (**Figure 2B**).

Earlier studies suggest that the mechanistic basis underlying the glucan matrix associated resistance phenotype is due to sequestration of antifungal by the matrix material away from the drug's cellular target [14]. We tested the biofilm sequestration capacity of the reference strain and the subset of glucan modification mutants, *bgl2*−/−, *xog1*−/−, and *phr1*−/− (**Figure 2C**). Each of the mutants sequestered less radioactive fluconazole than the reference strain, with the greatest defect observed for the *phr1*−/− biofilm (nearly 4-fold). The mechanistic reason for differences among the glucan modifying strains is not clear and is clearly an interesting area for further inquiry. This finding further links the glucan modifying enzymes and matrix glucan deposition to biofilm drug resistance.

### The impact of glucan modifying enzymes on cell wall composition and cell wall integrity

**Figure 3:**
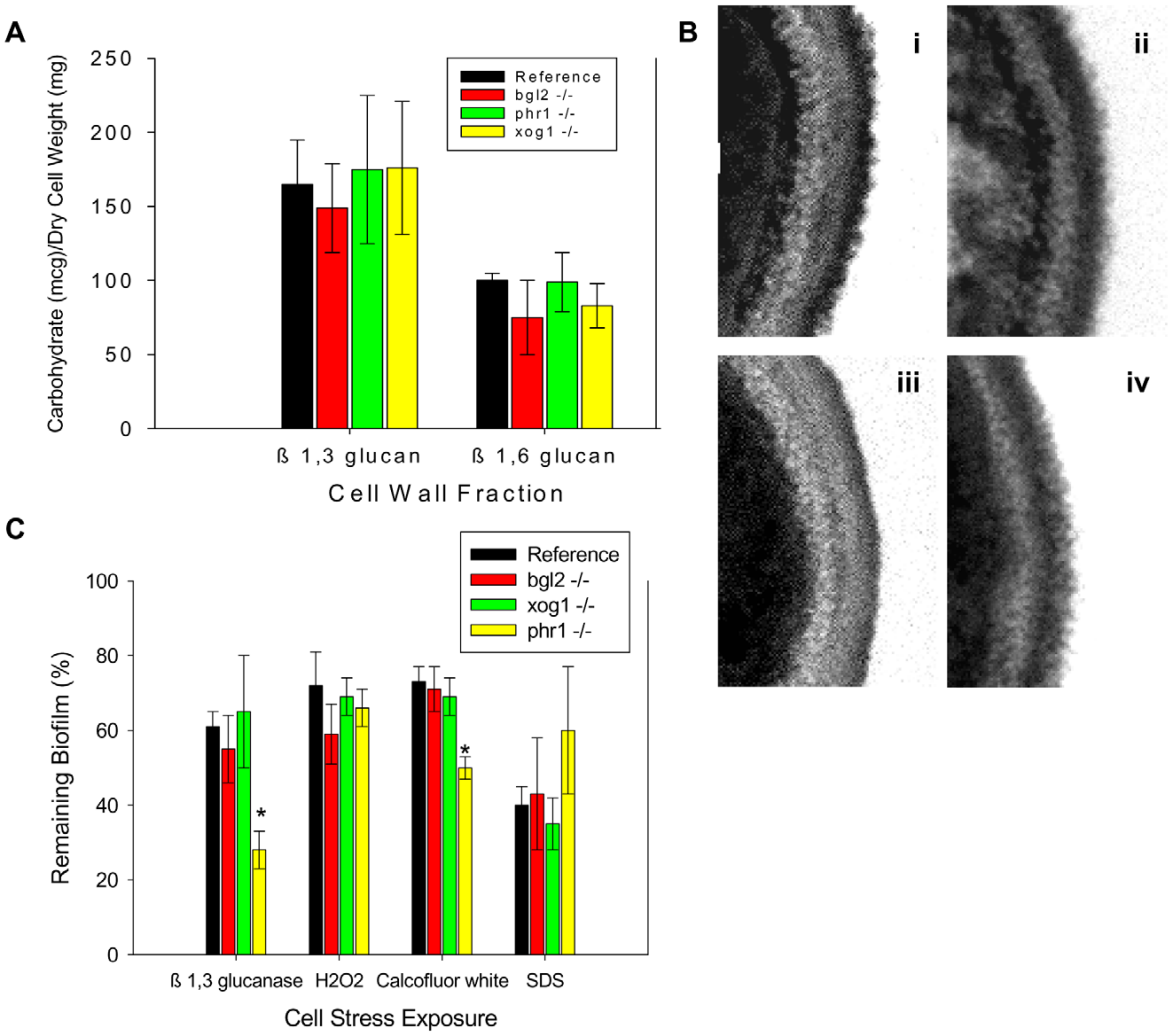
Impact of glucan modification enzyme mutants on cell wall composition and function of biofilm cells. (A) Cell walls from reference strain, *bgl2*−/−, *xog1*−/−, and *phr1*−/− mutant biofilms were isolated and fractionated by alkali treatment and enzymatic digestion. ANOVA with pairwise comparisons using the Holm-Sidak method was used to compare the β-1,3 glucan and β-1,6 glucan fractions among the strains *, p<0.05. Assays were performed in triplicate on two occasions. Standard deviations are shown. (B) Reference strain (i) and *bgl2*−/− (ii), *xog1*−/− (iii), and *phr1*−/− (iv) mutant biofilms were collected from 6-well polystyrene plates and imaged using TEM. Scale bars represent 0.25 um. (C) Reference strain and *bgl2*−/−, *xog1*−/−, and *phr1*−/− mutant biofilms were treated with serial dilutions of β-1,3 glucanase, H_2_O_2_, calcofluor white, or SDS. Compound impact was determined using an XTT reduction assay. Data are expressed as percent remaining biofilm compared to untreated controls. Standard errors are shown. ANOVA with pairwise comparisons using the Holm-Sidak method was used to compare the mutant strains at each drug concentration *, p<0.05.

Understanding the function of this subset of glucan modifying enzymes, Bgl2, Xog1, and Phr1, in cell wall construction and maintenance remains incomplete. We hypothesized that the cell wall of mutant strains with reduced matrix β-1,3 glucan may exhibit similar glucan reductions in the cell wall. Previous studies in a *phr1−/−* mutant show altered cell wall glucan and chitin content during planktonic growth [29]. We were surprised to find similar cell wall β-1,3 and 1,6 glucan content among the biofilm cells of this subset of glucan modifying mutants and the reference strain (**Figure 3A**). These results support a model in which the individual modification enzymes are dispensable for cell wall glucan production during biofilm growth, but are required for delivery of glucan from the cell to the extracellular matrix. The difference between the *PHR1* cell wall results in the planktonic and current biofilm studies further underscore a novel, biofilm specific role for this gene product.

Light microscopy and transmission electron microscopy (TEM) were used to inspect the mutant cell wall phenotypes. Light microscopy of the cells demonstrated a previously described abnormal hyphal morphology in the *phr1*−/− strain (data not shown) [35]. However, the other mutants appeared similar to the reference strain. By TEM, the yeast cell walls for each of the strains appeared quite similar in thickness and ultrastructural composition, consistent with the carbohydrate composition analyses (**Figure 3B**). The relative thickness of the cell wall of at least 50 images from each strain was quantified using ImageJ software. The average cell wall thickness for each strain was not significantly different from the reference strain (data not shown).

A parallel study of cell wall function was performed to assess the potential impact of the glucan modifying genes on the cell wall integrity pathway that has been shown to contribute to the biofilm formation and drug resistance mechanism [36], [37]. Susceptibilities to β -1,3 glucanase, hydrogen peroxide, SDS, and calcofluor white were similar among the *bgl2*−/−, *xog1*−/−, and the reference biofilms (**Figure 3C**). The *phr1*−/− strain exhibited hypersensitivity to β -1,3 glucanase and calcofluor white, and a relative resistance to SDS. The change in susceptibility to calcofluor white in these biofilm experiments is similar to that described for planktonic conditions [38]. These phenotypic screens suggest potential perturbation of the CWI pathway associated with *PHR1* disruption, but we did not detect a similar signal for the other glucan modifying mutants.

### Functional relationship between glucan modifying enzymes and Fks1p glucan production

**Figure 4:**
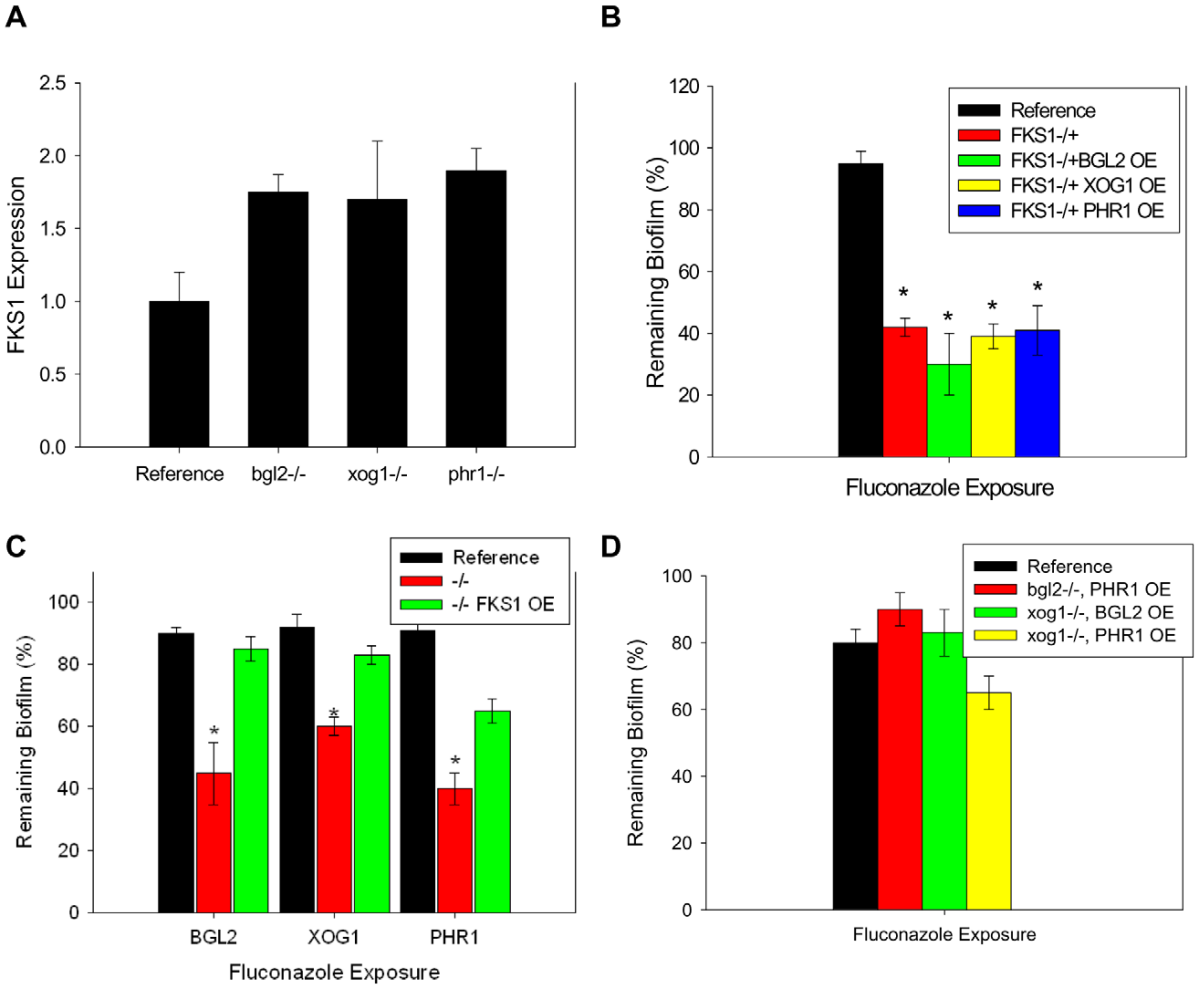
Relationship between β-1,3 glucan synthase and modification enzymes during biofilm growth. (A) RNA was isolated from reference and *bgl2*−/−, *xog1*−/−, and *phr1*−/− mutant biofilms. Real-time RT-PCR assays were used to measure transcript levels in triplicate. Data are shown as a normalized ratio of transcript in the mutant strain to that in the reference strain. (B) The glucan modifier genes, *BGL2*, *XOG1*, and *PHR1* were placed under the control of an inserted *TDH3* promoter for overexpression of these genes in the homozygous *FKS1*−/+ mutant. Biofilms were treated with serial dilutions of fluconazole for 48 h (250 µg/ml shown), and drug impact was determined using an XTT reduction assay. The * symbol indicates biofilm susceptibilities were significantly different (p value<0.008) based upon ANOVA with pairwise comparison. (C) The *FKS1* gene was placed under the control of an inserted *TDH3* promoter for overexpression of this genes in the *bgl2*−/−, *xog1*−/−, and *phr1*−/− mutants. Biofilms were treated with serial dilutions of fluconazole for 48 h (250 µg/ml shown) and drug impact was determined using an XTT reduction assay. (D) The glucan modifier genes, *BGL2*, *XOG1*, and *PHR1* were placed under the control of an inserted *TDH3* promoter for overexpression of these genes in each of the homozygous *bgl2*−/−, *xog1*−/−, and *phr1*−/− mutants. Biofilms were treated with serial dilutions of fluconazole for 48 h (250 µg/ml shown) and drug impact was determined using an XTT reduction assay. Data for all XTT assays above are expressed as percent reduction compared to untreated controls. Standard errors are shown. Student's *t* test was used for (C) and (D) to compare the mutant strains at each drug concentration *, p<0.05.

The β-1,3 glucan synthase has been shown as necessary for β-1,3 glucan production and development of biofilm matrix [16], [39]. We theorized that one or more of the glucan modification enzymes acts upon the β-1,3 glucan product of the synthase enzyme in a tightly controlled glucan delivery and matrix maturation of pathway. To explore this hypothesis we examined transcript abundance of the glucan synthase, *FKS1*, in the glucan modifier mutants. We reasoned that reduced delivery of glucan to the matrix may signal the cell to produce additional β-1,3 glucan which would be marked by an increase in the *FKS1* transcript. The FKS1 mRNA abundance results were consistent with the theory that matrix glucan levels influence the cell glucan production machinery. Transcript levels were elevated more than 1.5-fold in each of the modifier mutants (**Figure 4A**).

Additional testing of these relationships included a functional study of the impact of overexpression of the glucan modification genes, *BGL2*, *XOG1*, and *PHR1* in the *FKS1*−/+ heterozygote. This strain produces less matrix glucan and exhibits a biofilm antifungal drug susceptible phenotype [32]. We theorized that if the glucan modifier enzymes act upon the glucan product of Fks1p for matrix delivery, then overexpression of the modifiers would not repair the drug susceptibility defect in the *FKS1*−/+ background. Indeed, the overexpression of the glucan modifiers did not restore the wildtype biofilm resistance phenotype (**Figure 4B**). In a complementary experiment, we also examined the impact of overexpression of *FKS1* in the glucan modifier null−/− background. These manipulations restored the antifungal resistance phenotype to each of the modifier deletion mutants (**Figure 4C**).

### Functional relationship among the glucan modifying enzymes, Bgl2, Xog1, and Phr1

One simple explanation for these observations is a model in which the glucan modification enzymes provide complementary activity. Studies in the last several years have taught us that redundancy in the biofilm formation process is a common theme for other important functions, such as adherence [40], [41]. A second interpretation of the findings is a paradigm in which the glucan synthesis and modification pathways are distinct with regard to the biofilm matrix resistance mechanism. The suggestion of complementary activity for matrix delivery and drug resistance was further investigated by overexpression analysis of the glucan modifier genes in companion deletion mutant backgrounds and double knockout strains. We successfully introduced a *PHR1* overexpression allele into the *bgl2*−/− strain, and introduced a *BGL2* overexpression allele into *xog1*−/− and *phr1*−/− strains. We were unable to successfully introduce a *XOG1* overexpression allele in the *bgl2*−/− or *phr1*−/− strains. Similarly, we were unable to introduce the *PHR1* overexpression allele in the *xog1−/−* background. Biofilm susceptibility testing demonstrated restoration of the drug resistance phenotype associated with overexpression of a companion glucan modifier in the glucan modification mutant background for all strains tested (**Figure 4D**). These results are similar to those observed for in the glucan synthase mutant. We infer that the findings suggest a complementary relationship among the glucan modifiers.

**Figure 5:**
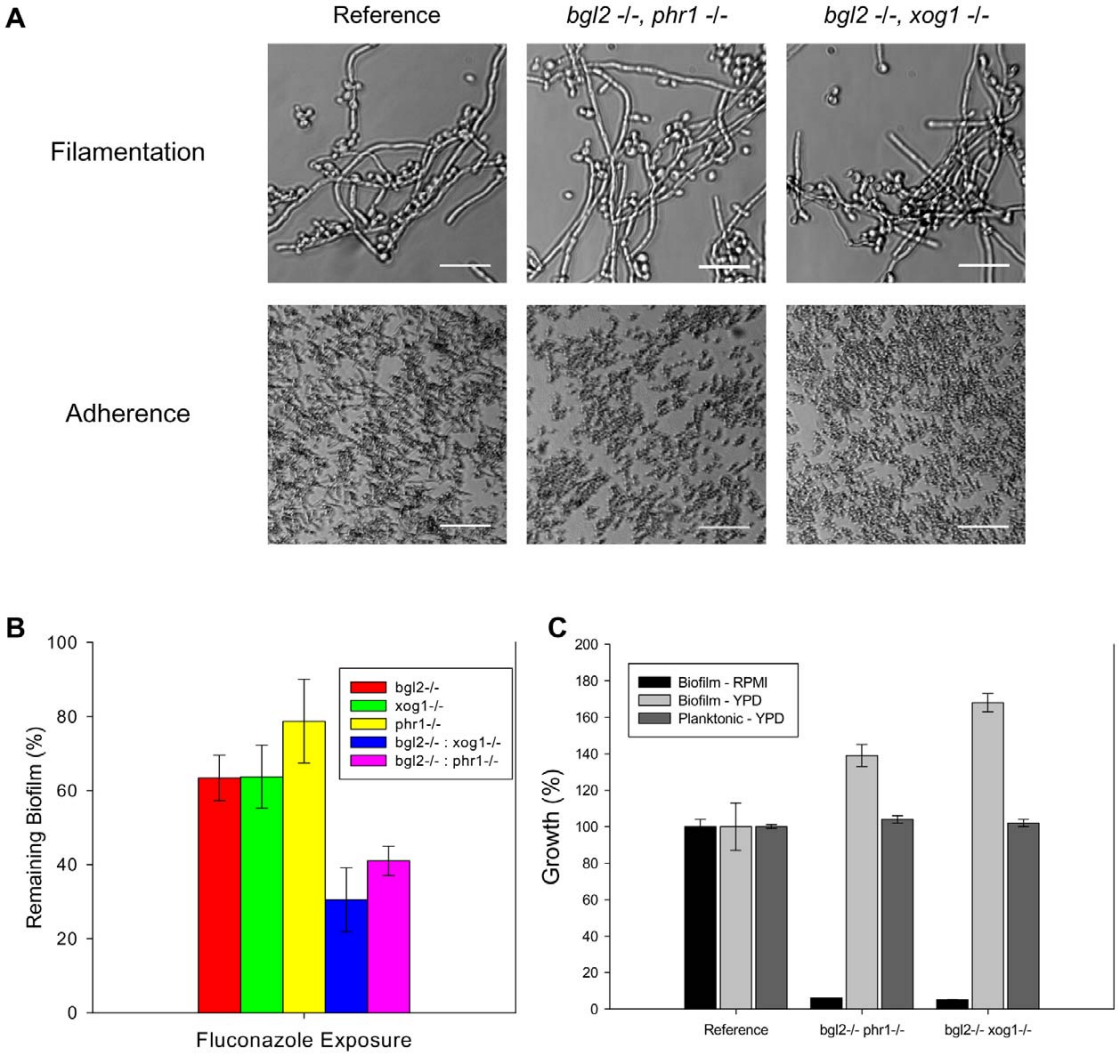
Analysis of glucan modifier double knockout strains. (A) The glucan modifier double knockout strains and the reference strain were assayed for filamentation in YPD. Representative images from light microscopy are shown (top row). The strains were also assayed for adherence to coverslips following 2 h incubation. Representative light micrographs are shown (bottom row). (B) The double knockouts were examined for overall biofilm growth in both YPD and RPMI by comparing the ODs of the untreated control with those of the reference strain in an XTT assay. Strains were also examined for relative planktonic growth in YPD using a turbidity endpoint. (C) The glucan modifier double knockout strains (*bgl2*−/− *phr1*−/− and *bgl2*−/− *xog1*−/−), the modifier single knockouts, and the reference strain were assayed for biofilm susceptibility to fluconazole (125 µg/ml shown) using the XTT assay as described above. Data for all XTT assays above are expressed as percent reduction compared to untreated controls. Standard errors are shown.

Additional examination of the association among the glucan modifiers included testing the impact of mutants in which two modifiers were disrupted. We were unable to construct the double knockout *xog1−/−*, *phr1−/−*, suggesting that loss of both of these genes may result in a non-viable mutant. For unclear reasons, the constructed double knockouts (*xog1−/−*, *bgl2−/− and bgl2−/−*, *phr1−/−*) demonstrated a growth defect in RPMI-MOPS under both biofilm and planktonic conditions, such that no biofilm could form in RPMI. They were, however, able to adhere to plastic and produce filaments in response to increased temperature when grown in YPD (**Figure 5A**). These double knockout strains also exhibited normal planktonic growth in YPD (**Figure 5B**). Therefore, we adapted the XTT biofilm drug susceptibility assay to include YPD media for comparison of double mutant and parent strains. In this assay, both of the double knockouts (*bgl2−/−*, *phr1−/− and xog1−/−*, *bgl2−/−*) demonstrate increased susceptibility to fluconazole when compared to their single modifier knockout parent strains (**Figure 5C**). While these strains produced relatively normal biofilms in the 96 well format, similar study with YPD in the larger format utilized for matrix composition analysis was insufficient in these strains. Thus, we were unable to reliably compare matrix glucan content. Although the observed RPMI growth defects and assay modification are limitations, the experiments suggest that deletion of two modification genes results in a further decline in matrix delivery. These findings support the theory that the modifiers act in parallel and can partially compensate for each other.

## Discussion

### Novel matrix delivery role for glucan modification enzymes

The extracellular matrix is critical for mature biofilm formation [42]. This material not only contributes to the adhesive nature of biofilm cells, but has been shown to protect the cells from antimicrobial agents and the host immune system as well [12], [30], [33], [43], [44]. Understanding the matrix components' production and delivery processes is one path for the development of effective biofilm therapies. A key constituent of the *C. albicans* matrix is β-1,3 glucan [16], [31]. Previous work identified an increase in cell wall glucan associated with biofilm growth [16]. Subsequent observations demonstrated the importance of the glucan synthase pathway for production of β-1,3 glucan in both the cell walls of biofilm cells and the extracellular matrix [32]. The predominant β-1,3 glucan synthase in *C. albicans* is encoded by *FKS1* [45]. Both the MAP kinase pathway and the transcription factor *ZAP1* have been identified as upstream components of the biofilm matrix production process [30], [31], [46]. However, the process of delivering glucan from the cell wall and the resulting mature biofilm matrix accumulation remained unknown. The present findings identify a novel role of several glucan modification enzymes for delivery of matrix glucan and other components to the cohesive extracellular matrix network.

The delivery enzymes from the current screen have been shown or suggested to act upon the β-1,3 glucan substrate. The function of each includes glucan hydrolysis and in some instances transfer and formation of new branch linkages. Previous studies in two unrelated bacterial pathogens, *Pseudomonas aeruginosa* and *Streptococcus mutans*, have demonstrated the importance of similar transferase enzymes for delivery of glucan to their biofilm matrices [43], [47]. Our glucan matrix and biofilm antifungal susceptibility screens point to a role for three genes, *BGL2*, *XOG1*, *and PHR1*. *BGL2* and *PHR1* encode glucanosyltransferases and *XOG1* encodes a β-1,3 exoglucanase [22], [27], [29]. Each of these genes has been shown to play a role in cell wall remodeling and specifically glucan chain elongation and cross-linking during planktonic cell growth for both *C. albicans* and *S. cerevisiae* [23]–[30]. Interestingly, each of the enzymes shown to impact matrix glucan delivery did not appear to impact the quantity of cell wall ultrastructure or β-1,3 glucan concentration. This suggests that these enzymes function specifically for matrix delivery, distinct from the cell wall assembly pathway during biofilm growth. One exception is *PHR1*. Disruption of this gene appeared to alter the cell wall integrity pathway during biofilm growth, based upon enhanced susceptibility to cell wall stress by calcofluor white. This observation is similar to that described for planktonic conditions [29]–[30].

Previous investigations found elevated transcript levels of *BGL2*, *XOG1*, and *PHR1* during in vivo biofilm growth compared to planktonic growth [18]. This biofilm associated upregulation is consistent with a role in a biofilm-specific function, such as matrix formation. The current studies identify a biofilm-specific pathway for these enzymes involving matrix delivery. One proposed mechanism is that the enzymes release and modify cell wall glucan for deposition in the extracellular space. An alternative explanation is that the enzymes act in the extracellular space, contributing to steric changes in glucan that are important for mature matrix organization and function. The enzymes Bgl2, Xog1, and Phr1 have been localized to the cell wall, supporting the hypothesis of cell wall activity. However, Bgl2 and Xog1 also contain secretion sequences providing feasibility for an extracellular function. Phr1 contains a GPI-linked tail, making it more likely to be tethered to the extracellular portion of biofilm cells. *Candida* biofilm proteomic analysis (our data not shown) identified Bgl2 Xog1, and Phr1 incorporated in the biofilm extracellular matrix consistent with an extracellular role. Enzyme isolation and further structural analysis of matrix components in parent and mutant strains may be an attractive strategy to differentiate between these matrix delivery functions.

### Link between the glucan synthase and biofilm matrix delivery

**Figure 6:**
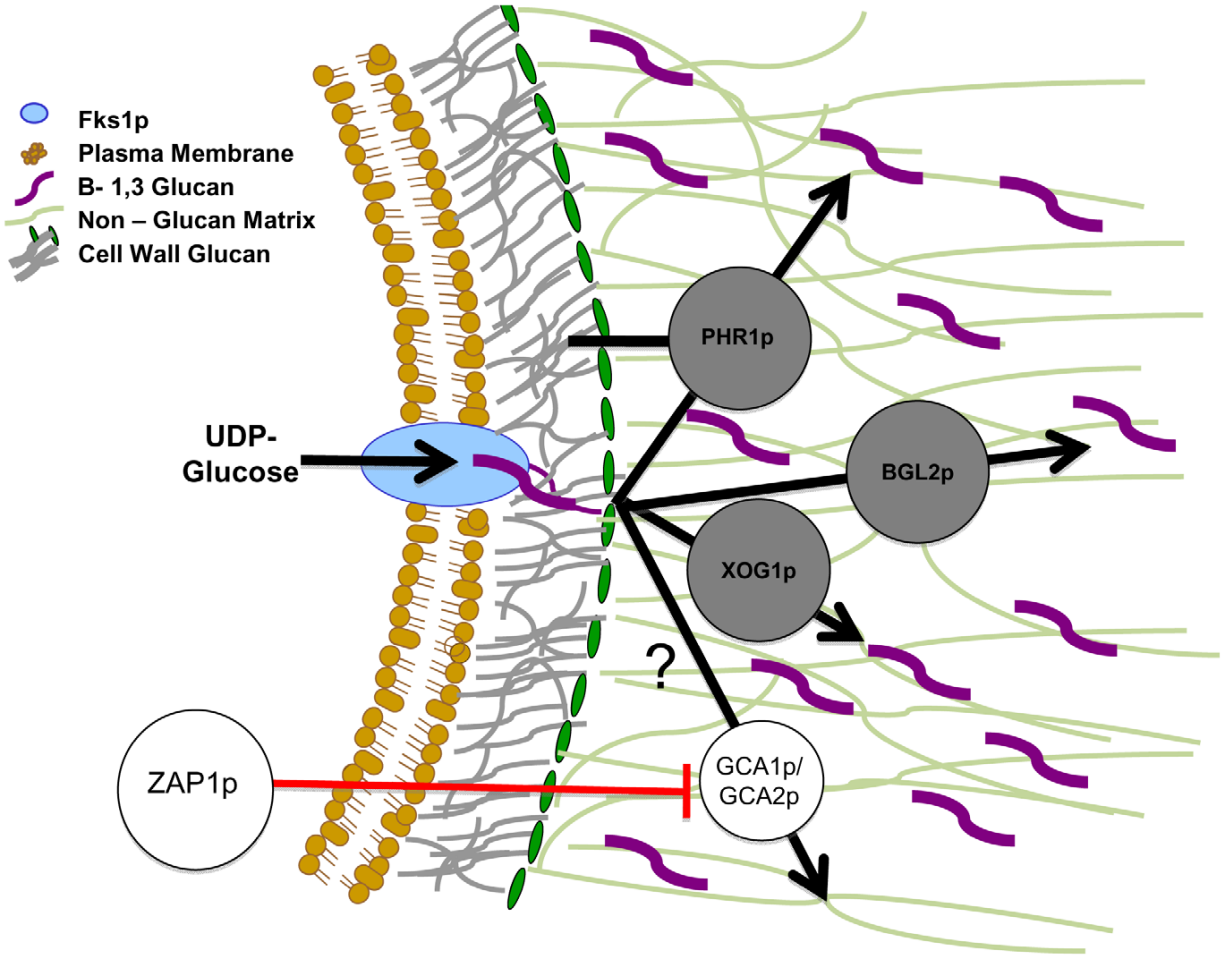
Proposed model for glucan matrix production and delivery to the biofilm matrix. The cell membrane-bound FKS1p is responsible for β-1,3 glucan production. This glucan is modified and incorporated into both the cell wall and the extracellular matrix. It is proposed that PHR1p, BGL2p, and XOG1p are responsible for the delivery and arrangement of β-1,3 glucans in the matrix and that they act independently of the cell wall arrangement pathway. We theorize PHR1p, BGL2p, and XOG1p act in a complementary fashion and independently of the ZAP1p matrix production pathway, which includes the glucoamylases GCA1p and GCA2p [31]. The glucan modification proteins are shown in the extracellular matrix based upon our matrix proteomic results. It is possible they are also located in the cell wall. We have not represented this possibility in the absence of these experimental results. Black arrows represent the proposed pathway taken by glucan from synthase product to the modified sugars that are incorporated into biofilm matrix. The circles represent production or modification enzymes that act upon carbohydrate substrates. Matrix glucans are highlighted in purple to distinguish from cell wall glucan (colored grey). Red lines represent inhibition of enzymes by transcription factors.

The known glucan modification function of these enzymes intuitively supports a model whereby matrix delivery is downstream of the primary β-1,3 glucan synthase encoded by *FKS1* (**Figure 6**). Transcriptional and functional analyses of our target gene overexpression strains support a pathway with partially redundant glucan modification enzymes that link to Fks1. We propose that the overexpression of glucan modifications enzymes is unable to compensate for disruption of *FKS1* due to the lack of available glucan substrate. Overexpression of *FKS1* partially restores the glucan mutant phenotype by over production of glucan substrate which is processed through parallel pathways of redundant glucan modification enzymes. Data derived from studying the double modifier mutants and the overexpression of modifiers in companion knockout strains also supports a degree of redundancy among the glucan modifiers. Furthermore, upregulation of the *FKS1* transcript in each of the enzyme modifier knockouts suggests feedback signaling for the cell to produce additional β-1,3 glucan during biofilm growth in the absence of glucan matrix delivery associated with each glucan modifier mutant.

We considered the possibility that the glucan modification pathway was under control of Zap1, a transcription factor known to function in matrix production. Surprisingly, review of previously reported global expression analysis of *ZAP1* did not identify altered expression of *XOG1*, *PHR1*, or *BGL2*. We confirmed the absence of differential mRNA abundance of these select glucan modification enzymes in the *ZAP1* mutant (data not shown). Thus, this work has identified a novel matrix glucan delivery pathway that is distinct from the previously described matrix-inhibitory pathway controlled by *ZAP1*. **Figure 6** shows a proposed model of the relationship of these matrix delivery enzymes to Fks1 and Zap1.

These studies show a novel biofilm matrix delivery pathway linked to the drug resistance phenotype. It is intriguing to consider the potential for drug target development designed specifically to identify enzyme inhibitor molecules. Because homologues are not present in the human genome, the likelihood of a safe pharmacologic anti-biofilm agent is promising. Additional work on the signaling and upstream genetic control of these enzymes promises to shed additional light on this important feature of biofilm formation.

## Methods

### Ethics statement

All animal procedures were approved by the Institutional Animal Care and Use Committee at the University of Wisconsin according to the guidelines of the Animal Welfare Act, The Institute of Laboratory Animal Resources Guide for the Care and Use of Laboratory Animals, and Public Health Service Policy.

### Media

Strains were stored in 15% (vol/vol) glycerol stock at −80°C and maintained on yeast extract-peptone-dextrose (YPD) medium with uridine (1% yeast extract, 2% peptone, 2% dextrose, and 80 µg/ml uridine) prior to experiments. *C. albicans* transformants were selected on synthetic medium (2% dextrose, 6.7% yeast nitrogen base [YNB] with ammonium sulfate, and auxotrophic supplements), or on YPD plus clonNat (2% Bacto peptone, 2% dextrose, 1% yeast extract, and 400 µg/ml clonNat [Werner Bioagents]) or on YPD plus 70 µg/ml hygromycin B (PhytoTechnology Laboratories). Prior to biofilm experiments, *C. albicans* strains were grown at 30°C in YPD and biofilms were grown in RPMI 1640 buffered with morpholinepropanesulfonic acid (RPMI-MOPS).

### Strains and strain construction

The *C. albicans* strains used in these studies are listed in **Table 1** and the genotypes in **Table S2 in Text S1**. Homozygous deletion strains were constructed from one of two parent strains, BWP17 or SN152. PCR product-directed gene deletion in the BWP17 background was performed as previously reported [48], [49]. Fusion PCR disruption cassettes were utilized to construct null strains in the SN152 background as previously described [50]. Complementation of mutant strains was performed using selection for arginine prototrophy as previously published [30], [51]. DNA cassettes of the entire gene as well as 1 kb up and downstream were amplified using PCR. The primers were designed to affix a BamHI site to the 5′ end of the DNA cassette and an AscI site to the 3′ end. Because *XOG1* had a BamHI cutting site within the gene, it was complemented using two AscI sites instead. Digested PCR products were ligated into the *E. coli* plasmid pC23, which carries ampicillin resistance for selection and encodes the *Candida dubliniensis* Arg4. Plasmids were linearized using PmeI and transformed using the lithium acetate protocol. All genotypes were verified by colony PCR using corresponding detection primers. Primers are listed in **Table S3 in Text S1**.

Overexpression of genes, *FKS1*, *XOG1*, *BGL2*, and *PHR1*, was accomplished by replacing the endogenous promoter of one allele with the promoter of *TDH3*, using the plasmid pCJN542 containing the *NAT1* – *TDH3* gene cassette as described previously [52]. Primers were designed with homology to the plasmid as well as to the promoter region of the targeted gene. This homology allowed for the entire cassette produced from the plasmid (including the *NAT1* gene and *TDH3* promoter) to be inserted into the promoter region of the gene of interest using homologous recombination, resulting in the gene now being driven by the highly active *TDH3* promoter. Transformants were selectively grown on YPD+clonNAT. All genotypes were verified by colony PCR.

Double deletion mutants were created in the SN152 background. The alleles for the first mutant were constructed by sequential replacement with the HygB^R^ and Nou^R^ resistance markers, respectively [53]. The second gene was disrupted by replacement of auxotrophic genes as described above [50]. The mutant strains were confirmed by colony PCR. The strain *xog1−/− : phr1−/−* could not be created.

### RNA isolation and real-time RT-PCR expression analysis

RNA was collected from biofilm cells grown in 6-well plates, as described below. RNA was purified using the RNeasy Minikit (Qiagen) and quantified using a NanoDrop spectrophotometer. TaqMan primer and probe sets designed using Primer Express (Applied Biosystems, Foster City, CA) for *ACT1*, *FKS1*, *BGL2*, *XOG1*, and *PHR1* are shown in **Table S4 in Text S1**. The QuantiTect probe reverse transcription-PCR (RT-PCR) kit (Qiagen) was used in an iQ5 PCR detection system (Bio-Rad) with the following program: 50°C for 30 min, initial denaturation at 95°C for 15 min, and then 40 cycles of 94°C for 15 s and 60°C for 1 min. Reactions were performed in triplicate. The expression of each gene relative to that of *ACT1* is presented. The quantitative data analysis was completed using the delta-delta *CT* method [54]. The comparative expression method generated data as transcript fold change normalized to a constitutive reference gene transcript (*ACT1*) and relative to the reference strain.

### *In vitro* biofilm model

Biofilms were grown in 6-well or 96-well flat-bottom polystyrene plates as previously described [51], [55]. The *C. albicans* inoculum (10^6^cells/ml) was prepared by growth in YPD with uridine overnight at 30°C, followed by dilution in RPMI-MOPS based on hemocytometer counts. For 6-well plates, 1 ml of culture was inoculated in each well. After a 60 min adherence period at 30°C, the non-adherent inoculum was removed and 1 ml of fresh medium (RPMIMOPS) was applied to each well. Plates were incubated at 37°C for 48 h on an orbital shaker set at 50 rpm. Medium was removed and fresh medium was added midway through the incubation period.

### *In vivo C. albicans* venous catheter biofilm model

A jugular vein rat central venous catheter infection model was used for *in vivo* biofilm studies [34]. *Candida* strains were grown to late logarithmic phase in YPD at 30°C with orbital shaking at 200 rpm. Following a 24 h conditioning period after catheter placement, infection was achieved by intraluminal instillation of 500 µl of *C. albicans* (10^6^cells/ml). After an adherence period of 6 h, the catheter volume was withdrawn and the catheter was flushed with heparinized saline. For drug treatment experiments, fluconazole (250 µg/ml) was instilled in the catheter after 24 h of biofilm growth. After a 24 h drug treatment period, the post treatment viable burden of *Candida* biofilm on the catheter surface was measured by viable plate counts on Sabouraud's dextrose agar (SDA) following removal of the biofilm by sonication and vortexing.

### *In vitro* biofilm and planktonic antifungal susceptibility testing

A tetrazolium salt XTT [2,3-bis-(2-methoxy-4-nitro-5-sulfophenyl)-2H-tetrazolium-5-carboxanilide inner salt] reduction assay was used to measure *in vitro* biofilm drug susceptibility [56], [57]. Biofilms were formed in the wells of 96-well microtiter plates, as described above. After a 6 h biofilm formation period, the biofilms were washed with phosphate-buffered saline (PBS) twice to remove non-adherent cells. Fresh RPMI-MOPS and drug dilutions were added, followed by additional periods of incubation (48 h). The antifungals studied included fluconazole at 4 to 1,000 µg/ml. Drug treatments were reapplied after 24 h, and plates were incubated for an additional 24 h. Following treatment with 90 µl XTT (0.75 mg/ml) and 10 µl phenazine methosulfate (320 µg/ml) for 30 min, absorbance at 492 nm was measured using an automated plate reader. The percent reduction in biofilm growth was calculated using the reduction in absorbance compared to that of controls with no antifungal treatment. Assays were performed in triplicate, and significant differences were measured by analysis of variance (ANOVA) with pairwise comparisons using the Holm-Sidak method.

The CLSI M27 A3 broth microdilution susceptibility method was used to examine the activities of fluconazole against planktonic *C. albicans* [58]. Endpoints were assessed after 24 h by visible turbidity.

### Biofilm biocide susceptibility testing

Agents with various mechanisms of action known to impact cell integrity were tested [37], [59]. A 96-well XTT assay, as described above, was used for measurement of the biofilm response to stress-inducing agents. The concentration required for a 50% reduction in XTT absorbance (50% effective concentration [EC50]) was recorded as the endpoint. Assays were performed in triplicate. The following concentration ranges were tested: calcofluor white, 0.2 to 200 µg/ml; β 1,3 Glucanase, 0.625 to 5 units/ml; H_2_O_2_, 25–200 µM; and sodium dodecyl sulfate (SDS), 0.001 to 2%.

### Biofilm SEM

In vitro biofilms were grown on sterile coverslips (Thermanox) in sterile 12 well plates and coated with 10 µl of human NaEDTA plasma each, which were dried at 30°C. 40 µl of yeast in RPMI, counted and diluted as in the biofilm models described above, was added to each coverslip for 60 min at 30°C. The initial inoculum was then removed and the plates incubated in 1 ml RPMI+MOPS+5% NaEDTA human plasma for 20 h at 37°C and 50 rpm on an orbital shaker. Media was replaced with 1 ml of fixative (4% formaldehyde, 1% glutaraldehyde in PBS) and coverslips were incubated at 4°C for 24 hours. The coverslips were then washed with PBS and treated with 1% osmium tetroxide for 30 min at ambient temperature. After a series of alcohol washes (30 to 100%), final desiccation was performed by critical-point drying. Coverslips were mounted, palladium – gold coated, and imaged in a scanning electron microscope (SEM LEO 1530) at 3 kV. The images were assembled using Adobe Photoshop 7.0.1.

### Biofilm cell TEM

*C. albicans* biofilms were grown on 6-well polystyrene plates for 48 h as described above. Cells were prepared for transmission electron microscopy (TEM) as previously described [30]. Following fixation in 4% formaldehyde and 2% glutaraldehyde, cells were postfixed with 1% osmium tetroxide and 1% potassium ferricyanide, stained with 1% uranyl acetate, dehydrated in a graded series of ethanol concentrations, and embedded in Spurr's resin. Sections (70 nm) were cut, placed on copper grids, poststained with 8% uranyl acetate in 50% methanol and Reynolds' lead citrate, and analyzed by TEM (Philips CM 120). The total cell and cell wall areas of 50 reference and mutant biofilm cells were measured using NIH Image J (http://rsbweb.nih.gov/ij/). The percentages of the cell wall area, defined as the cell wall area divided by the total cellular area, were calculated. Student's *t* test was used to determine statistical significance of differences between strains.

### Cell wall carbohydrate composition

Biofilms growing in 6-well plates for 48 h were washed with PBS and collected for cell wall carbohydrate analysis as previously described [16], [60]. Briefly, cells (5 mg dry cell weight) were washed with PBS and broken apart with glass beads. Isolated cell walls were alkali extracted for 60 min with 500 µl of 0.7 M NaOH at 75°C three times. The combined alkali-soluble supernatants were neutralized with 250 µl glacial acetic acid. Following neutralization, the alkali-insoluble pellet was digested with 100 U Zymolyase 20T (MP Biomedicals) at 37°C for 16 h. One half of the Zymolyase-soluble fraction was dialyzed (Slide-A-Lyzer dialysis cassette, 7,000-molecular-weight-cutoff [MWCO]; Pierce) to yield a β-1,6-glucan fraction. The β-1,3-glucan fraction was calculated as the difference between the total Zymolyase-soluble glucan and β-1,6-glucan fractions. The carbohydrate contents of each fraction were measured as hexoses by the phenol-sulfuric acid method and normalized for dry cell wall weight. ANOVA with pairwise comparisons (Holm-Sidak method) was used to determine statistical significance.

### Biofilm matrix collection and matrix_β-1,3 glucan measurements

The matrix β-1,3 glucan content was measured using a *Limulus* lysate based assay, as previously described [16]. Matrix was collected from *C. albicans* biofilms growing in the wells of 6-well polystyrene plates for 48 h. Biofilms were dislodged using a sterile spatula, sonicated for 10 min, and centrifuged 3 times at 4,500×*g* for 20 min to separate cells from soluble matrix material. Samples were stored at −20°C, and glucan concentrations were determined using the Glucatell (1,3)-β-D-glucan detection reagent kit (Associates of Cape Cod, MA) per the manufacturer's directions. Glucan concentrations were normalized for comparison across strains based upon viable biofilm burden using the XTT assay described above.

Matrix β-1,3 glucan was also measured using an ELISA assay. Biofilm was grown for 48 hours in 5×850 cm^2^ roller bottles (Corning, Thermo-Fisher) at 37°C. Biofilms were harvested into H_2_O using a sterile spatula then sonicated at 42 kHz for 20 min to dislodge the matrix. Next, biofilms were centrifuged 3×4,000 rpm for 20 min to separate the cells from the soluble matrix. The supernatant was lyophilized, dialyzed in a 3 kDa dialysis membrane (Spectra, Thermo-Fisher), and re-lyophilized to a powder. One mg of powdered matrix, dissolved in 1 ml of PBS was used as the sample in the ELISA assay and laminarin was used as a standard. A range of 1–1000 ng/ml of laminarin was used for the ELISA standard curve. 200 µl of 1 mg/ml matrix for each strain was assayed in triplicate. Plates were incubated overnight at 4°C, followed by blocking with 1% BSA for 45 min at ambient temperature. A 1∶2000 dilution of anti- β-1,3-glucan (BioSupplies Inc, Australia) was used as the primary antibody and a 1∶10,000 dilution of goat anti-mouse IgG-Biotin labeled [Sigma, Saint Louis] was used as a second antibody. Avidin-Peroxidase (Sigma) was used for detection.

### Accumulation of [H^3^] fluconazole into *C. albicans* biofilms

A radiolabeled fluconazole accumulation protocol was adapted for biofilm use as previously described [51], [61]. Biofilms were grown in 6-well plates, as detailed above. The biofilms were washed with sterile water twice. For stock solution preparation, radioactive [H^3^] fluconazole (Moravek Biochemicals; 50 µM, 0.001 mCi/ml in ethanol) was diluted 100-fold in water. The stock solution was then diluted 6-fold in RPMI-MOPS, and each biofilm well received a total of 600 µl of this medium to yield a total of 8.48×10^5^ cpm of [H3] fluconazole. After incubation for 30 min at 37°C and orbital shaking at 50 rpm, unlabeled (cold) 20 µM fluconazole in RPMI-MOPS was added and biofilms were incubated for an additional 15 min. Biofilms were then washed twice with sterile water, dislodged with a spatula, and collected as intact biofilms for scintillation counting. The biofilms were then disrupted by vortexing and sonication to separate cells and matrix. Following centrifugation, cells were separated from the soluble matrix material. Cells were subsequently disrupted by bead beating, and the intracellular and cell wall portions were collected by centrifugation. The fractions were then suspended in ScintiSafe 30% LSC cocktail (Fisher Scientific) and counted in a Tri-Carb 2100TR liquid scintillation analyzer (Packard). ANOVA was used to determine statistical significance of differences among strains.

### Biofilm disaggregation assay

Biofilms were grown using the 96 well microtiter model described above for 24 hours. Then, 90 µl of fresh media and 90 µl of serial 2 fold dilutions of the β-1,3 glucanase (Zymolyase - 20T, MP Biomedicals) in 0.9% NaCl was added to each well, with concentrations ranging from 5 U/ml to 0.625 U/ml. Plates were incubated at 37°C for 24 hours, at which point the media was removed and the biofilms were washed gently in 100 µl of PBS to remove any non-adherent cells. The plates were read using the XTT assay as described above. For comparison, a duplicate set of plates was spun at 3,000 RPM for 5 minutes before the media was removed on the final day. These biofilms were read via the XTT assay immediately, without washing, thus quantifying all living cells in each well to show whether β-1,3 glucanase at the concentrations used causes cell disaggregation or lysis.

## Supporting Information

Text S1 **Supplemental table legends.** Table S1 contains phenotypic results for all strains used in the studies. Table S2 contains the genotype for each strain used. Table S3 contains the primer sequences used for strain construction.
(DOCX)
