# Supplementary material for: A Candida Biofilm-Induced Pathway for Matrix Glucan Delivery: Implications for Drug Resistance
Source: PLoS Pathog. 2012 Aug 2;8(8):e1002848. doi: 10.1371/journal.ppat.1002848 (PMC3410897; doi:10.1371/journal.ppat.1002848)
Supplement: Text S1 — Supplemental table legends. Table S1 contains phenotypic results for all strains used in the studies. Table S2 contains the genotype for each strain used. Table S3 contains the primer sequences used for strain construction. (DOCX) [file ppat.1002848.s001.docx]

| **Strains** | **Systematic Name** | **Gene Name** | **Genotype** | **Description****** | **Biofilm**  **Formation*** | **Biofilm Resistance**** | **Matrix Glucan*** (pg/ml)** | **Planktonic MIC (µg/ml)** |
| --- | --- | --- | --- | --- | --- | --- | --- | --- |
| SN250 DAY185 | - | - | Reference |  | 100% | 100 | 1700 | 0.5 |
| FB63 HTT111 URZ5.2 | 19.4565 | BGL2 | -/- | β 1,3 glucanosyltransferase | 120% | 30% | 890 | 0.5 |
| GKO229 HTT117 | 19.2990 | XOG1 | -/- | Exo β 1,3 glucanase | 110% | 65% | 570 | 0.5 |
| KMR101 | 19.3829 | PHR1 | -/- | β 1,3 glucanosyltransferase | 75% | 40% | 190 | 0.25 |
| JEN152 | 19.2952 | EXG2 | -/- | Exo 1,3 beta glucosidase | 150% | 87% | 1400 | 0.25 |
| JEN120 | 19.6081 | PHR2 | -/- | β 1,3 glucanosyltransferase | 110% | 86% | 1800 | 0.5 |
| SCW11-U1 | 19.3893 | SCW11 | -/+ | β 1,3 glucosidase | 115% | 84% | 2000 | 1.0 |
| SPR1 | 19.2237 | SPR1 | -/- | Exo 1,3 β glucanase | 97% | 82% | 1400 | 0.5 |
| ENG1-U1 | 19.3066 | ENG1 | -/- | Endo 1,3 β glucanase | 80% | 80% | 1700 | 0.5 |
| JEN146 | 19.4668 | - | -/- | Glucosidase | 94% | 80% | 1600 | 1.0 |
| CU87-1 | 19.744 | GDB1 | -/- | Glucanotransferase | 100% | 77% | 1500 | 0.25 |
| I463-2 | 19.7339 | BGL22 | -/- | Glucanase | 95% | 76% | 1300 | 0.5 |
| GKO237 | 19.2237 | SPR1 | -/- | Exo 1,3 β glucanase | 90% | 76% | 1800 | 1.0 |
| ENG2-HM | 19.3417 | ENG2 | -/- | Endo 1,3 β glucanase | 90% | 78% | 1600 | 2.0 |
| JJH17 | 19.1779 | MP65 | -/- | Glucanase | 75% | 71% | 1700 | 0.5 |
| JEN153 | 19.1719 | SGA1 | -/- | Glucosidase | 110% | 70% | 1500 | 0.25 |

**Table S1. Select Strain Phenotypes.**

*Percent biofilm formation compared to reference strain. **Percent of biofilm remaining after exposure to fluconazole at 1000 µg/ml, ***Matrix β-1,3 glucan concentration normalized to biofilm burden, ****Based upon Candida or Saccharomyces Genome Database

**Table S2. Strain Genotypes.**

| Gene | Strain | Genotype | Ref |
| --- | --- | --- | --- |
| Reference | SN152 | *URA3 IRO1 arg4 his1 leu2*  *ura3::λimm434 iro1::* λimm434 *arg4 his1 leu2* |  |
| *phr1 -/-* | KMR101 | *URA3 IRO1 arg4 his1 leu2 phr1::C.d HIS1*  *ura3::λimm434 iro1::* λimm434 *arg4 his1 leu2 phr1::C.m LEU2* | This study |
| *phr1 -/-* | URZ302 | *URA3 IRO1 arg4 his1 leu2 phr1::HygB^R^*  *ura3::λimm434 iro1::* λimm434 *arg4 his1 leu2 phr1::Nou^R^* | This study |
| *phr1 -/-, +* | KMR148 | *URA3 IRO1 arg4 his1 leu2::PHR1::C.d ARG4 phr1::C.d HIS1*  *ura3::λimm434 iro1::* λimm434 *arg4 his1 leu2 phr1::C.m LEU2* | This study |
| phr1 *-/-, TDH3-FKS1* | HTT121 | *URA3 IRO1 arg4 his1 leu2 phr1::C.d HIS1* FKS1::pAgTEF1-NAT1-AgTEF1UTR-TDH3-PHR1  *ura3::λimm434 iro1::* λimm434 *arg4 his1 leu2 phr1::C.mLEU2 FKS1* | This study |
| *bgl2 -/-* | FB63-1 | *ura3::λimm434 arg4::hisG his1::hisG bgl2::URA3*  *ura3::λimm434 arg4::hisG his1::hisG bgl2::ARG4* | This study |
| *bgl2 -/-* | HTT111 | *URA3 IRO1 arg4 his1 leu2 bgl2::C.d HIS1*  *ura3::λimm434 iro1::* λimm434 *arg4 his1 leu2 bgl2::C.m LEU2* | This study |
| *bgl2 -/-* | URZ271 | *URA3 IRO1 arg4 his1 leu2 bgl2::HygB^R^*  *ura3::λimm434 iro1::* λimm434 *arg4 his1 leu2 bgl2::Nou^R^* | This study |
| *blg2 -/-, +BGL2* | HTT118 | *URA3 IRO1 arg4 his1 leu2::BGL2::C.d ARG4 bgl2::C.d HIS1*  *ura3::λimm434 iro1::* λimm434 *arg4 his1 leu2 bgl2::C.m LEU2* | This study |
| *bgl2 -/-, TDH3-FKS1* | HTT122 | *URA3 IRO1 arg4 his1 leu2 bgl2::C.d HIS1* FKS1::pAgTEF1-NAT1-AgTEF1UTR-TDH3-BGL2  *ura3::λimm434 iro1::* λimm434 *arg4 his1 leu2 bgl2::C.mLEU2 FKS1* | This study |
| *xog1 -/-* | GKO229 | *ura3::λimm434 arg4::hisG his1::hisG xog1::URA3*  *ura3::λimm434 arg4::hisG his1::hisG xog1::ARG4* | This study |
| *xog1 -/-* | HTT117 | *URA3 IRO1 arg4 his1 leu2 xog1::C.d HIS1*  *ura3::λimm434 iro1::* λimm434 *arg4 his1 leu2 xog1::C.m LEU2* | This study |
| *xog1 -/-* | URZ304 | *URA3 IRO1 arg4 his1 leu2 xog1::HygB^R^*  *ura3::λimm434 iro1::* λimm434 *arg4 his1 leu2 xog1::Nou^R^* | This study |
| *Xog1 -/-, +* | HTT125 | *URA3 IRO1 arg4 his1 leu2::XOG1::C.d ARG4 xog1::C.d HIS1*  *ura3::λimm434 iro1::* λimm434 *arg4 his1 leu2 xog1::C.m LEU2* | This study |
| *Spr1 -/-* | SPR1-KO#1 | *ura3::λimm434 arg4::hisG his1::hisG spr1::URA3*  *ura3::λimm434 arg4::hisG his1::hisG spr1::ARG4* | This study |
| *Eng1 -/-* | ENG1-U1-A1 | *ura3::λimm434 arg4::hisG his1::hisG eng1::URA3*  *ura3::λimm434 arg4::hisG his1::hisG eng1::ARG4* | This study |
| *Orf 19.4668 -/-* | JEN146 | *URA3 IRO1 arg4 his1 leu2 orf 19.4668::C.d HIS1*  *ura3::λimm434 iro1::* λimm434 *arg4 his1 leu2 orf 19.4668::C.m LEU2* | This study |
| *gdb1 -/-* | CU87 - 1 | *ura3::λimm434 arg4::hisG his1::hisG gdb1::URA3*  *ura3::λimm434 arg4::hisG his1::hisG gdb1::ARG4* | This study |
| *Spr1 -/-* | GKO237 | *ura3::λimm434 arg4::hisG his1::hisG spr1::URA3*  *ura3::λimm434 arg4::hisG his1::hisG spr1::ARG4* | This study |
| *Bgl22 -/-* | I463-2 | *ura3::λimm434 arg4::hisG his1::hisG bgl22::URA3*  *ura3::λimm434 arg4::hisG his1::hisG bgl22::ARG4* | This study |
| *Eng2 -/-* | ENG2 #2HM | *ura3::λimm434 arg4::hisG his1::hisG eng2::URA3*  *ura3::λimm434 arg4::hisG his1::hisG eng2::ARG4* | This study |
| *Mp65 -/-* | JJH17 | *ura3::λimm434 arg4::hisG his1::hisG mp65::URA3*  *ura3::λimm434 arg4::hisG his1::hisG mp65::ARG4* | This study |
| *Cas5 -/-* | JEN153 | ura3::λimm434 *arg4::hisG his1::hisG* cas5::URA3  ura3::λimm434 arg4::hisG his1::hisG *cas5::ARG4* | [[59](#_ENREF_59)] |
| *FKS1 - /+* | FKS1/fks1Δ | ura3::λimm434 *arg4::hisG his1::hisG* fks1::URA3  ura3::λimm434 arg4::hisG his1::hisG *FKS1* | [[62](#_ENREF_62)] |
| *FKS1 - /+, TDH3 – BGL2* | HTT128 | ura3::λimm434 *arg4::hisG his1::hisG* fks1::URA3 BGL2::pAgTEF1-NAT1-AgTEF1UTR-TDH3-FKS1  ura3::λimm434 arg4::hisG his1::hisG *FKS1 BGL2* | This study |
| *FKS1 - /+, TDH3 – PHR1* | HTT131 | ura3::λimm434 *arg4::hisG his1::hisG* fks1::URA3 PHR1::pAgTEF1-NAT1-AgTEF1UTR-TDH3-FKS1  ura3::λimm434 arg4::hisG his1::hisG *FKS1 PHR1* | This study |
| *FKS1 - /+, TDH3 – XOG1* | HTT132 | ura3::λimm434 *arg4::hisG his1::hisG* fks1::URA3 XOG1::pAgTEF1-NAT1-AgTEF1UTR-TDH3-FKS1  ura3::λimm434 arg4::hisG his1::hisG *FKS1 XOG1* | This study |
| *bgl2 -/-, TDH3-PHR1* | HTT139 | *URA3 IRO1 arg4 his1 leu2 bgl2::C.d HIS1* PHR1::pAgTEF1-NAT1-AgTEF1UTR-TDH3-FKS1  *ura3::λimm434 iro1::* λimm434 *arg4 his1 leu2 bgl2::C.m LEU2* PHR1 | This study |
| *xog1 -/-, TDH3-BGL2* | HTT136 | *URA3 IRO1 arg4 his1 leu2 xog1::C.d HIS1 BGL2*::pAgTEF1-NAT1-AgTEF1UTR-TDH3-FKS1  *ura3::λimm434 iro1::* λimm434 *arg4 his1 leu2 xog1::C.m LEU2 BGL2* | This study |
| *phr1 -/-, TDH3-BGL2* | HTT134 | *URA3 IRO1 arg4 his1 leu2 bgl2::C.d HIS1 BGL2*::pAgTEF1-NAT1-AgTEF1UTR-TDH3-FKS1  *ura3::λimm434 iro1::* λimm434 *arg4 his1 leu2 bgl2::C.m LEU2 BGL2* | This study |
| *bgl2 -/-,xog1 -/-* | URZ290 | *URA3 IRO1 arg4 his1 leu2 bgl2::HygB^R^ xog1::C.d. HIS1*  *ura3::λimm434 iro1::* λimm434 *arg4 his1 leu2 bgl2::Nou^R^ xog1::C.m.LEU2* | This study |
| *bgl2 -/-,phr1 -/-* | URZ296 | *URA3 IRO1 arg4 his1 leu2 bgl2::HygB^R^ phr1::C.d. HIS1*  *ura3::λimm434 iro1::* λimm434 *arg4 his1 leu2 bgl2::Nou^R^ phr1::C.m.LEU2* | This study |

**Table S3. Primers for Strain Creation.**

|  | **Function** | **Primers** |
| --- | --- | --- |
| *BGL2* | Knockout | Upstream F: 5’ - TTTATTTTCTTGCTTGTACC  Upstream R: 5’ - cacggcgcgcctagcagcggAGTGGGTATAAGAATGAAGT  Auxotrophic marker F: 5’ – ccgctgctaggcgcgccgtgACCAGTGTGATGGATATCTGC  Auxotrophic marker R: 5’ – gcagggatgcggccgctgacAGCTCGGATCCACTAGTAACG  Downstream F: 5’ – gtcagcggccgcatccctgcACTTGGATTTTTCTTATTTC  Downstream R: 5’ – GTCAAAGCAATGTATAAAAG  Nested Fusion F: 5’ – AATTGCTACTGCGGAGGAAGG  Nested Fusion R: 5’ – TTCCAGGAAAAGGTGTTTGG  Upstream Check F: 5’ – AAGAAAAGTAAAATCTGACG  Downstream Check R: 5’ – TAATTACTCGTTCACTTTGA  Internal Check F: 5’ - TTTGAAGCTGAAAAGGAAGC  Internal Check R: 5’ - TTCAAAGTTTTGTCGGATTGC |
| *BGL2* | Complement | BamHI F: 5’ - GTAGGATCCTTTTTCTTCTCCTGCTGCTG  AscI R: 5’ - GGTGGCGCGCCCAATTCCTTCTGGGGATCAA |
| *BGL2* | Overexpression | TDH3 OE F: 5’ – TAATTCTTGTCCCAAAATAGACCCAAGTTCTAGATTTGGAGATGCACGATGTTGCTATAAATAGTACTCACATGAAAAACATACCCAAATTAGGAAGTCAATCAAGCTTGCCTCGTCCCC  TDH3 OE R: 5’ - TACCGTCATCATTTTTAACACCAAGGTTGAAAGCCAAATCACCCATGGCGGCAACTGAAGTAAGAACAGTTGCGAGAGTAGTCAAGAATTTGATTTGCATATTTGAATTCAATTGTGATG  OE determinant F: 5’ - GCCACAGGGAATGTATTGAT  ClonNAT marker R: 5’ - GAAACAACAACGAAACCAGC |
| *PHR1* | Knockout | Upstream F: 5’ - CATTTTTAACCAACCCTTGTT  Upstream R: 5’ - cacggcgcgcctagcagcggTTTTTGGCTTCAACCTGTAG  Auxotrophic marker F: 5’ – ccgctgctaggcgcgccgtgACCAGTGTGATGGATATCTGC  Auxotrophic marker R: 5’ – gcagggatgcggccgctgacAGCTCGGATCCACTAGTAACG  Downstream F: 5’ – gtcagcggccgcatccctgcCCGATATGAAGGGTTCTGTT  Downstream R: 5’ – CAGTGATGGATTCAAAAGCA  Upstream Check F: 5’ – CAAATCATTCCACCTTGAAA  Downstream Check R: 5’ - AGCAGAAAGAGGAAAGCTGA  Internal Check F: 5’ - AAACAACCCAGAATGGAACT  Internal Check R: 5’ - GCACTGGATTTCTTGTCTTG |
| *PHR1* | Complement | BamHI F: 5’ – AAGGATCCTTCCATCTTTATAACCCAAATGAAA  AscI R: 5’ - AAGGCGCGCCGCTGATGAAAGTGAAACTAAGGAAG |
| *PHR1* | Overexpression | TDH3 OE F: 5’ - CGATTCCAGTCCAATACAATAGTTGTCACAAAACACGAAATACAATACTATTTTATTCTAATAATAGT TTTGTTTTTTTCTTTCTTTTATTTCGTCTTACATCAAGCTTGCCTCGTCCCC  TDH3 OE R: 5’ - TACCAACAACTTCAACTGGTGGAGTGGACGATTCAAACTTGGCTAAAGTTAATGAAAAGAGTGTGGCAAATGTAACCAATGATTTGATTAATGAATACATATTTGAATTCAATTGTGATG  OE determinant F: 5’ – CGGTCAAGTTTTCGGCTAAG  ClonNAT marker R: 5’ - GAAACAACAACGAAACCAGC |
| *XOG1* | Knockout | Upstream F: 5’ - TCCACCCCTGTTAAAATCCA  Upstream R: 5’ - cacggcgcgcctagcagcggACCGCAAATTGGAAAAATCG  Auxotrophic marker F: 5’ – ccgctgctaggcgcgccgtgACCAGTGTGATGGATATCTGC  Auxotrophic marker R: 5’ – gcagggatgcggccgctgacAGCTCGGATCCACTAGTAACG  Downstream F: 5’ – gtcagcggccgcatccctgcCCACTTGAAAATCTACCAAGC  Downstream R: 5’ – GTTTTTCTTTGTCGCGTCGT  Nested Fusion F: 5’ – GCTTTGTTGATCCAGTTTCAG  Nested Fusion R: 5’ – GGCTGTGCGATTCTAGAGTC  Upstream Check F: 5’ – AAGAAAAAGGGCAAGGAAAAA  Downstream Check R: 5’ – CTGAAGTCGTTGCCAGTTGT  Internal Check F: 5’ - AGGAGGCGGACATAATGTTG  Internal Check R: 5’ - AGCCAAAGACTTGGAAAGCA |
| *XOG1* | Complement | AscI F: 5’ - ATAGGCGCGCCGAAAAATAAAAGAGCCCACACG  AscI R: 5’ - TCAGGCGCGCCGTTTTTCTTTGTCGCGTCGT |
| *XOG1* | Overexpression | TDH3 OE F: 5’ - GCAAATTGATTTTTAAAACACTTTACACACATTTCGGAATTTTCAAGTAGGTTTTAGCTTTCTACAATTTGTTAATATAAAAGTGCAGAAAGTCCACCCCATCAAGCTTGCCTCGTCCCC  TDH3 OE R: 5’ - ATTTCAAGTTTCCATTTGGTTTAAATGGATTAGAAATAACCAGGGCTTTAACAAATTCAAGCAATAATATAAATACCGATGATGTTAAGATAAATGATAAATTTGAATTCAATTGTGATG  OE determinant F: 5’ – AAGAAAAAGGGCAAGGAAAAA  ClonNAT marker R: 5’ - GAAACAACAACGAAACCAGC |
| *FKS1* | Knockout | Described in [[62](#_ENREF_62)] |
| *FKS1* | Overexpression | Described in [[62](#_ENREF_62)] |
